# Natural Polymer-Based Coatings for Animal-Derived Products: A Review of Applications, Functionality, Characterization, and Challenges

**DOI:** 10.3390/foods14132255

**Published:** 2025-06-26

**Authors:** Márcio Vargas-Ramella, Noemí Echegaray, Paulo Cezar Bastianello Campagnol, José Manuel Lorenzo

**Affiliations:** 1 Centro de Educação Superior da Região Sul–CERES, Departamento de Ciências Biológicas, Universidade do Estado de Santa Catarina–UDESC, Laguna 88790-000, Santa Catarina, Brazil; 2 Centro Tecnológico de la Carne de Galicia, 32900 Ourense, Spain; noemiechegaray@ceteca.net (N.E.); jmlorenzo@ceteca.net (J.M.L.); 3 Centro de Ciências Rurais–CCR, Departamento de Tecnologia e Ciência dos Alimentos, Universidade Federal de Santa Maria (UFSM), Santa Maria 97105-900, Rio Grande do Sul, Brazil; paulo.campagnol@ufsm.br; 4 Área de Tecnología de los Alimentos, Facultad de Ciencias de Ourense, Universidad de Vigo, 32004 Ourense, Spain

**Keywords:** biodegradable packaging, edible film, biopolymer coating, shelf-life, active packaging, bioactive compounds, sustainable material, food

## Abstract

The global demand for sustainable packaging and animal-derived products’ perishability emphasizes the urgent need for biodegradable alternatives to petroleum-based materials (i.e., synthetic polymers or plastic). This narrative review explores the recent advancements in natural polymer-based coatings, comprising ingredients such as polysaccharides, proteins, and lipids, as well as their combination as multifunctional strategies for preserving meat, dairy, seafood, and eggs. These coatings act as physical barriers and can carry bioactive compounds, enhancing oxidative and microbial stability. Particular attention is placed on the structure-function relationships of biopolymers, their characterization through advanced techniques (e.g., Fourier Transform Infrared spectroscopy—FTIR, Scanning Electron Microscope—SEM, Differential Scanning Calorimetry—DSC, and Thermogravimetric analysis—TGA), and their functional properties (e.g., antimicrobial and antioxidant efficacy). Notably, food matrix compatibility is pivotal in determining coating performance, as interactions with surface moisture, pH, and lipids can modulate preservation outcomes. While several formulations have demonstrated promising results in shelf-life extension and sensory quality preservation, challenges remain regarding coating uniformity, regulatory compliance, and scalability. This narrative review highlights current limitations and future directions for the industrial application of these sustainable materials, aiming to link the gap between laboratory success and commercial feasibility.

## 1. Introduction

The global food industry faces a dual challenge: meeting rising demand for high-quality animal-derived products (i.e., meat, poultry, eggs, dairy, and seafood) while addressing the environmental burden of conventional plastic packaging. Despite the implementation of ambitious policy commitments by various governments, projections suggest that annual plastic emissions could rise to as much as 53 million metric tons by 2030 [1]. Recent advancements in bioeconomy frameworks highlight bio-based edible coatings—derived from renewable polysaccharides, proteins, and lipids—as biodegradable alternatives that align with the European Green Deal’s 2030 targets for sustainable packaging [2].

The urgency of this transition is magnified by the inherent perishability of animal-derived products, which are highly susceptible to spoilage due to their moisture-rich matrices, unsaturated lipid profiles, and nutrient density. These factors accelerate microbial proliferation, oxidative rancidity, and protein denaturation. For example, fresh beef can undergo lipid oxidation rates exceeding 3.0 μmol malondialdehyde (MDA)/kg (minimum threshold for the rancidity perception) after a few days under refrigeration [3]. On the other hand, poultry fresh meat can exhibit microbial loads of pathogens such as *Salmonella* spp., *Listeria monocytogenes*, *Campylobacter jejuni*, and *Clostridium perfringens* surpassing Colony Forming Unit—CFU/g limits to consumers, even in different packaging (air, modified atmosphere, under vacuum) and temperatures (0 °C, 4 °C, and 10 °C) storage conditions [4]. Traditional preservation methods, such as synthetic antioxidant additives and petroleum-based vacuum packaging, contribute to environmental pollution and introduce health risks through chemical migration [5]. For this reason, emerging studies have focused on demonstrating that natural-based polymer coatings, such as chitosan coating, can reduce the use of petroleum-based plastic to preserve food products. For instance, Yaghoubi et al. [6] found that chitosan (1%) with wormwood (*Artemisia fragrans*) essential oil, positively reduced total viable count (TVC), coliforms, molds, and yeasts contamination in poultry while maintaining their physicochemical and sensorial properties. In addition, the versatility of chitosan-based edible coatings in food preservation was further demonstrated by Faisal et al. [7]. Their study investigated a composite coating incorporating oil palm-derived liquid smoke and turmeric extract to extend the shelf life of mackerel (*Scomberomorus commerson*). The coating significantly reduced Total Volatile Basic Nitrogen (TVB-N) and TVC with sensory analysis confirming retained acceptability.

Therefore, natural polymers—polysaccharides, proteins, and lipids—serve as foundational matrices for edible coatings, addressing both environmental and preservation challenges in animal-derived products. Polysaccharides like chitosan [8] and alginate [9] were widely studied for their abundance, tunable barrier properties, and adaptability to functional enhancements. Protein-based systems, including gelatin and whey isolates, offer mechanical robustness while enabling the bioactive incorporation of essential oils to preserve pork flavor and taste [10], and cheese safety [11], respectively. Lipid blends like beeswax have been utilized to optimize structural homogeneity and moisture barriers in composite films, as demonstrated in wheat gluten matrices [12].

On the other hand, these coatings’ performance depends critically on ternary interactions between biopolymers, additives (e.g., essential oils, nanocellulose), and food matrices, which vary with pH, ionic strength, and molecular compatibility. Therefore, rigorous evaluation of coating efficacy is essential to translate these innovations into practical solutions. Current limitations in the commercial viability of biodegradable food packaging films, particularly regarding inadequate gas permeability and degradation rates, necessitate the development of optimized analysis to evaluate their feasibility [13]. For this reason, it is essential to strategically combine tests for evaluating the physical and mechanical properties of the films. Among these techniques, the following list of methods can offer a film’s characterization after its elaboration to support lab-scale innovation to industrial adoption:

- Structural and molecular characterization with Fourier-Transform Infrared Spectroscopy—FTIR (e.g., to identify functional groups and their molecular interaction/network to evaluate the correct manufacture of a film) [14] and Raman spectroscopy (e.g., as complementary insights to FTIR and other techniques) [15];
- Thermal stability with Thermogravimetric Analysis—TGA (e.g., to evaluate thermal degradation temperatures in cellulose biofilms) [16] and Differential Scanning Calorimetry—DSC (e.g., to characterize thermal transitions) [17];
- Mechanical performance with tensile tests by determining Young’s modulus—E (MPa), tensile strength -TS (MPa), and elongation at break—EAB (%) (e.g., whey protein-zein blends) [18];
- Barrier and surface properties with Water Vapor Permeability—WVP (e.g., to quantify moisture barrier efficacy (g·mm/m^2^·day·kPa)) [19];
- Surface morphology with a Scanning Electron Microscopy (SEM) (e.g., to visualize microstructure homogeneity and cracks) [9] and Transmission Electron Microscope (TEM) (e.g., for structural characterization) [20];
- Functional efficacy of antimicrobial and antibiofilm coatings against *Salmonella* and *Escherichia coli* isolated in meat using minimum inhibitory concentration (MIC) technique [21];
- Antioxidant capacity with total phenolic content (TPC), 2,2-diphenyl-1-picrylhydrazyl—DPPH, 2,2′-azinobis(3-ethylbenzothiazoline-6-sulfonic acid)—ABTS Radical Scavenging of active films (e.g., to evaluate antioxidant activity and antioxidant release kinetics) [22], FRAP (Ferric Reducing Antioxidant Power) (e.g., to quantify electron-donating capacity) [21], and the thiobarbituric acid reactive substances (TBARS) or peroxide value (PV) assays (e.g., to track secondary oxidation during storage [23];
- Coating compatibility to evaluate the interaction of the tested films with the food matrices (e.g., by simulated storage trials testing shelf-life extension and sensory analysis to assess consumer acceptance via hedonic scales—odor, texture, appearance) [16,24].

Therefore, this narrative review critically examines the multifunctional capacity of natural-based polymers to replace petroleum-based counterparts, emphasizing mechanistic insights into green polymer self-assembly, structure-function relationships, and bioactive compounds delivery. By addressing critical challenges—such as hydrophilic polymer matrices, batch-to-batch variability in biopolymers, and heterogeneous raw material feedstocks—these coatings offer a viable pathway to mitigate food spoilage while aligning with planetary health objectives. Additionally, characterization techniques enable precise quantification of barrier properties, antimicrobial efficacy, and oxidative stability, ensuring compliance with global regulatory standards. The present study aimed to provide scalable and recommendable preservation systems that harmonize industrial scalability with food matrix applications with extended shelf life. Different from previous papers published that broadly discuss polymers in food packaging, the present review uniquely focuses on natural polymer-based coatings specifically applied to animal-derived products. This includes a detailed evaluation of their chemical composition, functional performance, and instrumental characterization, and regulatory aspects. The integration of the analytical methods combined with a focus on coating compatibility with diverse animal-based matrices such as meat, dairy, seafood, and eggs, offers a comprehensive and application-specific perspective not previously addressed.

## 2. Natural Polymers: Categories of Coatings According to Composition

Natural polymer-based coatings are primarily categorized according to their utilization method in edible coatings and biofilms. Edible coatings are thin and adherent layers prepared from biological materials applied directly to food surfaces, forming a solidified coating (e.g., by immersion and spray). On the other hand, biofilms (also called edible films) are freestanding structures formed from the evaporation of the solvent used to prepare the edible coating and utilized to encapsulate food products [9,10,13].

Therefore, edible coatings and biofilm’s polymers can be classified by their origin into categories as follows:

- Proteins: gelatin, whey protein [25], casein, zein (maize prolamin) [26], and soy protein [27]
- Polysaccharides: chitosan (deacetylated chitin from crustacean shells), carrageenan (from red algae), agar (Gelidium/Gracilaria), pectin, cellulose derivatives (carboxymethyl cellulose (CMC) and cellulose nanofibers (CNF)) [28], starch (maize, potato, tapioca) [26]
- Lipids: beeswax, carnauba wax, and fatty acids [26]
- Microbially synthesized: xanthan gum (*Xanthomonas campestris*) [29], pullulan (*Aureobasidium pullulans*) [30], and bacterial cellulose (*Gluconacetobacter*, *Komagataeibacter xylinus*) [16]
- Monomer polymers: polylactic acid (PLA), and polyhydroxyalkanoates (PHA) [31]
- Composite systems: polysaccharide-protein (e.g., chitosan-gelatin) [32], polysaccharide-lipid (e.g., alginate-beeswax), nanocomposites (ginger nanocomposites embedded in hydrogel matrices) [33], cellulose nanocrystals, microbially synthesized-polysaccharide (e.g., pullulan-chitosan) [30], and crude bioactive algal extract (*Spirogyra* sp.)–chitosan [34]

Among all these groups, natural polymer-based coatings have emerged as a promising and sustainable approach to improve animal-derived foods’ quality, safety, and shelf-life. These biodegradable materials form protective barriers limiting moisture loss, lipid oxidation, and microbial contamination, reducing spoilage and extending product freshness [35,36]. In parallel, their use reduces dependence on petroleum-based packaging and synthetic food additives. Their versatility enables application across a broad range of products (including meat, eggs, dairy, and seafood) where they enhance microbiological and physicochemical stability. Furthermore, these coatings provide an effective delivery system for natural antioxidants and antimicrobials, supporting clean-label formulations. They represent a viable alternative to conventional preservation and packaging systems within the animal product sector by aligning with current consumer preferences and environmental goals.

In addition to the above discussion, incorporating natural agents into edible packaging using polymer matrices has demonstrated potential to mitigate foodborne pathogens and spoilage. Among the natural polymers utilized for packaging alternatives, with or without these additives, polysaccharides (e.g., chitosan and alginate) dominate research efforts due to their abundance, gel-forming capacity, tunable barrier properties, and ability to improve their functional properties with additives incorporation. Sun et al. [8] optimized chitosan’s antimicrobial activity by incorporating natamycin using cherry tomatoes to extend their shelf-life. The authors found that the film’s thickness, transparency, water vapor transmittance rate (WVTR), TS, and EAB were improved. Additionally, the authors suggested that their findings can be widely applied in dairy products to decrease oxidation, weight loss, and hardness. In line with this, Vargas-Ramella et al. [9] assessing alginate as edible coating—a natural polysaccharide derived from brown marine algae *Phaeophyceae*—evaluated alginate coating cross-linked with CaCl_2_ on *Mugil liza* fillets during storage. Authors found this packaging beneficial as an antimicrobial agent and in decreasing TVB-N parameters to avoid food spoilage.

Beyond polysaccharides, gelatin and whey protein isolates offer mechanically robust matrices for bioactive encapsulation. Gelatin, a protein-based material, is widely used for edible coatings due to its excellent film-forming ability, biodegradability, clarity, and compatibility with biological systems. Incorporating bioactive compounds—such as essential oils or plant extracts—into gelatin-based coatings can significantly improve their antioxidant and antimicrobial performance. Haoxin Li et al. [10] found that incorporating ginger (*Zingiber officinale*) essential oil into gelatin-based coatings has effectively preserved the flavour and taste quality of pork meat. Zandona et al. [11] evaluated the bioactive properties of the olive (*Olea europaea*) leaf extract incorporated into a whey protein isolate coating of cheese. The olive leaf-enriched coating retention of total phenols and flavonoids contributed to improve its antioxidant properties, reducing microbial counts without compromising cheese safety and quality.

On the other hand, it is important to consider that polymer-based coating efficacy depends fundamentally on ternary interactions between the base biopolymer, functional additives, and food matrix components, with variations in pH, ionic strength, and molecular compatibility potentially altering performance. Therefore, this comprehensive analysis of these interactions through rheological, spectroscopic, and microbiological assays is critical for formulation optimization. Padilla et al. [37] evaluated the effect of cellulose nanocrystals on the gelatin-matrix composite films and concluded that the various structural levels that act on gelatin matrices (and other films) are complex and do not imply synergism in their properties. Consequently, further investigation is needed to understand these interactions to modulate their interactions depending on their desired application.

Complementing these systems, lipid-based blends such as beeswax and composite systems (e.g., polysaccharide-protein, polysaccharide-lipid, nanocomposites, and cellulose nanocrystals) are part of the most studied natural-based polymers as edible coatings. Echegaray et al. [12], in their review, highlighted several relevant studies that exemplify the application of lipid incorporation via emulsification to enhance the functional properties of protein-based films. In particular, they discussed formulations involving wheat gluten–wheat bran cellulose matrices combined with varying concentrations (5–20%) of lipids such as paraffin wax, beeswax, and vegetable oils. Among these, beeswax was identified as particularly effective in improving the structural homogeneity and water vapor barrier properties of the coatings, with optimal performance observed at intermediate lipid concentrations.

Lastly, it is important to consider that these coating categories have been extensively explored in recent years, although their classification may vary depending on the authors and research approaches. Moreover, most studies have concentrated on fruit and vegetable applications, while investigations involving animal-based food products remain relatively limited. The effectiveness of natural coatings is closely linked to their specific formulation and the interaction with the food matrix, where factors such as surface morphology and moisture content play a key role [9].

## 3. Coatings Characterization

### 3.1. Chemical Composition: Structural and Molecular Characterization (FTIR, FE-SEM, and XRD)

The bio-film’s chemical components (i.e., structural and molecular characterization) can be evaluated using FTIR, Field-Emission Scanning Electron Microscopy (FESEM or FE-SEM), and X-ray Diffraction (XRD).

FTIR is a non-destructive analytical technique that identifies molecular vibrations in response to infrared radiation, generating a spectrum that reveals the functional groups and chemical bonds within a material [33]. By measuring absorption/transmission at specific wavelengths (400–4000 cm^−1^), FTIR provides insights into molecular interactions, cross-linking mechanisms, and structural modifications in natural polymer-based coatings. Practical applications in evaluating coatings can include: verification of cross-linking detecting molecular bonding [14], additive-polymer interactions by identifying molecular compatibility and functional group identification (e.g., ZnO nanoparticles with propolis-resinous substance collected by honey bees *Apis mellifera* L.—for meat packaging capped with polyphenolic and biomolecules of mushroom extract) [30], quality control and structural degradation under storage conditions. In addition, FTIR analysis can be combined with the following FE-SEM, XRD, TS, and biodegradability analysis to evaluate data consistency.

The FE-SEM is an advanced imaging technique that uses a focused electron beam to generate high-resolution morphological appearance with nanoscale surface morphology and compositional data. Unlike conventional SEM, FE-SEM employs a field-emission electron source, enabling superior resolution (<1 nm) and reduced sample charging [38]. The FE-SEM coatings’ evaluation can offer a surface morphology analysis indicating nanoscale features such as pores, cracks, and lipid/protein phase separation as well as the composite homogeneity by visualization of the dispersion of additives (e.g., irregular-shaped nanoparticles) within polymer matrices (e.g, pullulan/pectin [39] or pullulan/chitosan-based [30] composite). In addition, this technique offers a cross-sectional analysis that evaluates coating thickness uniformity and layer adhesion (e.g., chitosan-gelatin bilayers). FE-SEM can also be a part of the degradation assessment, pointing to structural changes during films’storage (e.g., antimicrobial activity) [40] and nanoparticle alignment (e.g., pullulan content) [39].

XRD is an important assessment to determine the morphology and crystallinity of biofilms with the aim of packaging material. The XRD is a non-destructive analytical technique that characterizes materials’ crystallographic structure and phase composition by measuring the diffraction patterns of X-rays scattered by atomic planes. XRD quantifies crystallinity, identifies polymorphic forms, and detects nanocomposite interactions in natural polymer-based coatings by analyzing angles and diffraction intensities. Crystallinity determines polymers’ amorphous-to-crystalline ratio (%), which governs barrier and mechanical properties. Nanocomposite characterization identifies nanoparticle dispersion (e.g., nanoparticles) via diffraction peak broadening [14,37,41,42].

### 3.2. Thermal and Mechanical Properties

#### 3.2.1. Thermal Stability

Thermal stability analysis evaluates a material’s resistance to decomposition or structural changes under controlled temperature conditions, providing critical insights into processing compatibility, storage resilience, and functional performance. Techniques such as TGA and Differential Scanning Calorimetry (DSC) are widely used to characterize thermal transitions (e.g., glass transition, melting, degradation) in natural polymer-based coatings. These analyses are useful for evaluating coatings in relation to identifying temperature thresholds for material degradation, guiding processing parameters (e.g., extrusion, drying), and glass transition temperature [17].

The DSC technique measures heat flow differences between samples and reference material under controlled temperature conditions. The DSC measures are critical for assessing flexibility and brittleness under storage conditions. This analysis can evaluate interactions between biopolymers and natural preservatives regarding thermal volatility and stability of additives such as essential oils [43] or isolated compounds like vanillin [44]. Furthermore, thermal stability assessment can evaluate degradation onset temperatures for processing compatibility while determining additive compatibility by assessing phase separation or molecular interactions between polymers and additives [45].

On the other hand, TGA is a technique that measures the mass loss of a material as a function of temperature under controlled atmospheric conditions. It can quantify thermal decomposition, moisture content, and additive stability [46]. This method identifies the temperature at which material degradation begins, guiding processing limits (e.g., extrusion, drying) [33]. The TGA can also quantify moisture and volatile content by measuring initial mass loss (10–600 °C) to assess water absorption or residual solvents [46].

#### 3.2.2. Mechanical Performance: Tensile Testing (TS, EAB, and E), and Rheology

Tensile testing is a mechanical analysis method that quantifies a material’s response to uniaxial stress, determining key parameters such as the TS, EAB, and E (expressed as Pa, MPa or GPa). These tests are performed via standardized protocols (e.g., ASTM standards methods D882-02 [47] or D638 [48]) and generate stress-strain curves to determine parameters. These tests can quantify resistance to tearing or cracking during handling, enabling the evaluation of (i) alternative polymers as replacements for conventional plastics (e.g., chitosan-based polyimine vitrimer [49] and (ii) natural additives incorporated into polymer matrices (e.g., rosemary essential oil [50] or red beet extract [51] on mechanical performance. Identifying trade-offs between TS and flexibility facilitates comparisons between natural coatings and synthetic counterparts while guiding industrial processing parameters, such as extrusion temperatures, to mitigate mechanical failure during packaging operations.

The TS is the maximum stress a material can withstand under uniaxial tensile loading before failure, which can be determined using the Texture Analyzer equipment. For natural polymer-based coatings, this parameter is critical for assessing mechanical durability, flexibility, and suitability for food packaging applications [52]. For example, the TS can quantify resistance to tearing or cracking during handling (e.g., vacuum packaging of food) [53], evaluate the impact of plasticizers (e.g., glycerol) [8] or reinforcements (e.g., Japanese rice vinegar) [54] on mechanical performance, and identify trade-offs between strength and flexibility. This technique can also compare natural coatings vs. conventional plastics and guide industrial processing parameters to prevent mechanical failure during high-speed packaging.

The EAB is a critical mechanical property that measures the ductility of natural polymer-based coatings, defined as the percentage increase in length that a material undergoes before fracture under tensile stress. For edible coatings applied to perishable animal-derived products, EAB reflects the coating’s ability to withstand mechanical deformation during handling, transportation, and storage without cracking or delaminating [55]. High EAB values indicate flexibility and resilience, which are essential for maintaining barrier integrity on irregular or dynamic food surfaces such as seafood (e.g., spider crab *Maja crispata* [56] and flounder *Paralichthys orbignyanusi* [57]) and chicken fillets [58].

The E, defined as the ratio of stress to strain within the elastic deformation region (E = σ/ε), measures a material’s stiffness and structural integrity. For natural polymer-based coatings, this parameter is critical for assessing flexibility, durability, and suitability for food packaging applications [59]. This modulus indicates resistance to deformation under stress, influencing coating flexibility during handling (e.g., beef packaging) [60] or how an environment can alter their mechanical behavior (e.g., UV radiation [61]. It also detects phase separation or poor interfacial adhesion and compares natural coatings to synthetic counterparts [62].

Roy et al. [30] evaluated TS, EAB, and E of an edible coating for meat packing and noted that the incorporation of propolis into the films increased its thickness. This effect was attributed to high-molecular-weight polyphenols in propolis, which may occupy the spaces between polymer chains, causing them to separate further. Additionally, this observation suggests a lack of chemical interaction between propolis and the polymer matrix.

Lastly, rheology is the study of the flow and deformation behavior of materials under applied stress, providing the viscoelastic properties, gelation kinetics, and processing characteristics of fluids and solids [63]. For edible films, rheological analysis can quantify parameters such as storage modulus (G′), loss modulus (G″), complex viscosity (η*), and yield stress, which govern coating application, adhesion, and structural stability [64]. Its practical applications in evaluating coatings include viscoelastic behavior determination of the balance between elastic (solid-like) and viscous (liquid-like) properties that represent the coatings` adhesion and flexibility [33,65]. The gelation kinetics by tracking cross-linking or sol-gel transitions (e.g., by-products for pectin extraction) to optimize extraction times and industrial application are also important parameters [66]. This method can also offer viscosity reduction data under shear stress, ensuring that coatings can be sprayed or brushed onto food surfaces without clogging equipment [67]. Moreover, rheology assays can identify the minimum stress required to initiate flow (i.e., yield stress), critical for preventing coating sagging on vertical surfaces and thermal stability in temperature ramps, and assess melt resistance (or denaturation thresholds [53].

### 3.3. Barrier and Surface Properties

#### 3.3.1. Barrier Film’s Properties: Biodegradability, Moisture Content, Swelling Index, and WVTR

The biodegradability refers to the ability of a material to decompose into natural substances (e.g., CO_2_, H_2_O, biomass) through enzymatic or microbial action under specific environmental conditions. For natural polymer-based coatings, biodegradability analysis evaluates their environmental compatibility and ensures they align with circular economy principles by avoiding persistent waste [51]. This analysis is critical for validating sustainability claims and compliance with global regulations (e.g., EU 10/2011 [68] and ASTM D6400 [69]).

Key methods for biodegradability assessment include biodegradability in vegetable compost or soil burial tests, in which coatings are buried in soil under controlled humidity/temperature, with periodic monitoring of mass loss and structural disintegration to simulate degradation in terrestrial environments (e.g., compostable packaging) [14,51,70,71]. Another measurement includes aerobic/anaerobic respirometry measuring CO_2_ or CH_4_ evolution during microbial decomposition following ISO standards [72] to quantify mineralization rates in composting facilities or landfills. Aquatic degradation tests can also be performed by submerging coatings in freshwater/seawater to assess disintegration and microbial colonization [73] to validate marine biodegradability for seafood packaging. Lastly, the compostability validation ensures that coatings meet industrial composting standards (e.g., ASTM D6400: >90% degradation in 180 days [69]) and eco-toxicity screening that confirms decomposition byproducts (e.g., oligomers, additives) are non-toxic to soil/water ecosystems.

For instance, for the soil burial application in bio-film tests, Choudhary et al. [71] buried samples at a depth of 2 cm and a distance of 3 cm apart and weighed (Wi). Weighing the test sample on an analytical balance (accuracy 0.00001 g) periodically before and after degradation testing, the authors used the data to calculate the weight loss (3 days). To calculate the percentage of weight loss, the following equation is used:

$$Biodegradability=\frac{Wi-Wf}{Wf}\times 100$$

where Wi and Wf are the initial and final weight of the sample after 3 days, respectively. The bio-degradation of the synthesized bio-film can be also investigated using already discussed FTIR technique by comparing the spectra of the bio-film at different stage (i.e., days)

Moisture content quantifies the water content within a material, expressed as the ratio of water mass to the total mass of the coating. This parameter is critical for assessing hygroscopicity, stability, and functional performance for natural polymer-based coatings, as excessive moisture can compromise barrier properties, accelerate microbial growth, or reduce mechanical integrity [74]. Regarding moisture content, tests such as the moisture retention, the water solubility (WS), and the WVP of the films are the most commonly employed. Its practical applications in evaluating coatings for hygroscopicity assessment by determining the coating’s tendency to absorb atmospheric moisture, influencing shelf-life and food product stability [75,76]. For instance, the barrier performance correlation to high moisture content in hydrophilic polymers such as gelatin can increase WVP, reducing efficacy in humid environments [22,62].

Concerning mechanical stability, moisture can decrease TS and increase microbial growth risk through mold or bacterial proliferation. Protocols to achieve target moisture levels for scalability can be assessed as follows:

$$Moisture\;retention\left(\%\right)=\frac{Wwet-Wdry}{Wdry}\times 100$$

where Wdry represents the initial weight of the dry bio-film and Wwet represents the weight of the bio-film after moisture adsorption [14].

Solubility refers to the ability of a material to dissolve in a specific solvent (e.g., water, ethanol, or food simulants) under defined conditions. For natural polymer-based coatings, solubility analysis evaluates their stability in aqueous or lipid environments, influences their barrier functionality, and determines their suitability for specific food applications (e.g., moisture-sensitive meats or water-resistant cheese coatings) [53]. Practical applications in evaluating coatings include their barrier performance by determining resistance to dissolution in high-moisture or acidic foods (e.g., fresh seafood, dairy). In addition, a “controlled release” can assess solubility-driven release of bioactive compounds (e.g., antioxidants, antimicrobials) or guide solvent selection for coating application (e.g., dipping or spraying method) [35]. As discussed above, this technique can also be combined with environmental degradation analysis to predict solubility in composting or marine environments.

As a practical approach to assess film solubility, the procedure can be adapted as follows [16]: each film under evaluation is cut into small pieces measuring approximately 20 mm × 20 mm and thoroughly dried in an oven at 110 °C to determine the initial dry weight (Wi). Subsequently, the dried samples are immersed in distilled water and agitated for 24 h at 30 °C in a sealed beaker. After this period, the remaining film fragments are carefully removed, rinsed to eliminate surface residues, and dried again at 100 °C to obtain the final dry weight (Wf). The solubility of the films is then determined using the following formula:

$$Solubility\left(\%\right)=\frac{Wi-Wf}{Wi}\times 100$$

Swelling index (SI) quantifies the ability of a material to absorb a solvent (typically water or food simulants) under controlled conditions, expressed as the percentage increase in mass or volume. For natural polymer-based coatings, this parameter is critical for evaluating hydrophilicity, water resistance, and structural stability in humid environments or when applied to high-moisture food products [42]. In relation to the hydrophilicity assessment, it can determine affinity for water absorption, influencing barrier properties and moisture retention. When evaluating cross-linking efficacy, it evaluates the success of chemical or physical cross-linking (e.g., Ca^2+^ in alginate) by measuring reduced swelling. SI can offer how hydrophobic additives (e.g., agarose/gum neem/nanohydroxyapatite/polyoxyethylene sorbitan monooleate) mitigate swelling when assessing additive compatibility. In addition, food matrix interaction can be predicted with the coating stability on high-moisture foods (e.g., fresh meat, cheese) under storage conditions and pH, as well as its biodegradability correlation, i.e., highly swollen coatings may degrade faster due to increased water penetration [14]. The swelling capacity of a film can be obtained as follows [71]:

$$Swelling\;index\left(\%\right)=\frac{Ws-Wd}{Wd}\times 100$$

where Ws represents the weight achieved after solubilization and Wd represents the initial weight of the film.

The WVTR quantifies the mass of water vapor that permeates through a material per unit area and time under specific temperature and humidity conditions, typically expressed in grams per square meter per day (g/m^2^/day). WVTR is a critical parameter for coatings to evaluate moisture barrier efficacy, directly influencing the shelf-life and quality of perishable animal-derived products (e.g., meats, dairy, seafood) by controlling dehydration, microbial growth, and lipid oxidation [50,77]. As practical applications in evaluating coatings can be measured to determine the coating’s ability to retain moisture in high-water-activity foods or prevent humidity ingress in dry products. In addition, it can also be utilized to evaluate the formulation optimization to guide the incorporation of hydrophobic additives and environmental adaptability to predict coating stability (e.g., refrigerated vs. tropical storage).

The WVTR is useful to estimate real-world performance of coatings under defined conditions (e.g., 25 °C, 80% relative humidity—RH) but depends on material thickness and environmental factors (temperature, humidity gradient) [50]. The methodology to calculate this feature normally utilizes a gravimetric cup method described by the ASTM E96 [19] with the coating sealed over a desiccant-filled cup and placed in a controlled humidity chamber. Weight gain (water vapor ingress) is measured over time with the following formula:

$$WVTR=\frac{∆W}{A\times t}$$

where ΔW is the weight change (g), A the coating area (m^2^), and t the time (days).

On the other hand, the WVP quantifies the intrinsic ability of a material to transmit water vapor normalized for thickness and vapor pressure gradient. Expressed in grams·millimeter per square meter·day·kilopascal (g·mm/m^2^·day·kPa), WVP compares the moisture barrier performance of coatings independent of their thickness. The WVP analysis is essential to assess their suitability for preserving perishable animal-derived products (e.g., meats, cheeses, seafood) by mitigating moisture loss or gain [41,51,54]. Among its practical applications in evaluating coatings the 3 main following data can be obtained: (i) material comparison of moisture barrier efficacy across different polymers (e.g., nopal mucilage vs. orange pectin) and thicknesses [41] (ii) additive optimization by evaluating the impact of hydrophobic additives (e.g., propolis [30] or microalgae [56]) on moisture barrier enhancement; and (iii) processing parameter guidance to informs drying, extrusion, or casting conditions to minimize defects (e.g., pores, cracks) that increase WVP [78]. Nanoparticles typically improve WVP properties by occupying interchain spaces and creating tortuous diffusion pathways for water molecules. For instance, propolis incorporation has consistently enhanced barrier properties due to its hydrophobic constituents, reducing film hygroscopicity [30].

Finally, assessing a material’s hydrophobicity is essential for evaluating its resistance to water and determining its appropriateness in packaging exposed to liquids or humid environments [52]. This is typically measured using the water contact angle (θ), where values above 90° indicate hydrophobic behavior, although in some cases, angles as low as 65° may also be considered acceptable. Biodegradable coatings with hydrophobic properties—especially those created through electrospinning techniques using modified biopolymers—present a promising eco-friendly alternative to traditional plastic packaging [18].

#### 3.3.2. Surface Morphology: SEM and Atomic Force Microscopy (AFM)

The SEM is a high-resolution imaging technique that employs a focused beam of electrons to scan the surface of a sample, generating detailed topographical and morphological information. By detecting secondary or backscattered electrons emitted from the sample’s surface, SEM produces magnified images with exceptional depth of field and spatial resolution, typically ranging from micrometers to nanometers. This method is particularly valuable for visualizing natural polymer-based coatings’ structural integrity, surface roughness, porosity, and homogeneity [9,53]. Considering the practical applications in evaluating coatings, SEM analysis plays a critical role in assessing the functional performance and structural quality of edible coatings derived from polysaccharides, proteins, and lipids, as well as if the coating additives (e.g., ZnO nanoparticles) were uniformly applied [79].

The SEM key applications include evaluating the uniformity and homogeneity assessment by the continuity of coating layers, identifying defects such as cracks, pinholes, or uneven thickness that compromise barrier properties. For example, alginate coating cross-linked with CaCl_2_ on films requires uniform surfaces to block oxygen and moisture effectively, and SEM imaging helps optimize formulation parameters [9]. In addition, this technique can identify phase compatibility in hybrid formulations of composite coatings, such as protein-lipid or polysaccharide-nanocomposite systems. SEM visualizes the distribution of reinforcing components (e.g., curcumin/cellulose microcrystals in chitosan films), ensuring cohesive integration critical for mechanical strength and barrier efficacy [80]. SEM can also evaluate the impact of drying methods [81] and post-treatment processes (e.g., refrigerated storage) [82,83] significantly influence coating morphology by tracking structural changes.

Another surface morphology analysis, the AFM, is a high-resolution imaging technique that employs a sharp nanoscale probe mounted on a flexible cantilever to scan a sample’s surface. By measuring the interaction forces between the probe and the surface, AFM generates three-dimensional topographic maps with sub-nanometer resolution. Unlike SEM, AFM can operate in air or liquid environments, eliminating the need for conductive coatings or vacuum conditions. This technique provides quantitative data on surface roughness, adhesion, and mechanical properties, making it ideal for characterizing soft, hydrated, or delicate coatings [17,54,80].

Considering its practical applications in coatings, AFM is widely used to investigate the nanoscale structural features of edible coatings. It offers insights critical to their functional performance by surface roughness quantification, i.e., AFM measures parameters that influence barrier properties. For instance, smoother polysaccharide films (e.g., alginate) reduce WVP, while lipid-reinforced coatings with controlled roughness may enhance hydrophobicity [53]. In addition, AFM micrographs detect phase separation or agglomeration in composite coatings as well as corroborate the FE-SEM data (e.g., the incorporation of propolis into pullulan/chitosan composite films helps maintain their surface roughness while enhancing functional properties) [30]. Variations in surface stiffness or adhesion maps reveal uneven distribution of ingredients added to coatings, such as cellulose microcrystals in chitosan films [80] or oil droplets in coatings formulated with sodium alginate [54] or gelatin-chitosan [17]. The generated images can guide formulation adjustments for a more uniform barrier performance. This is critical for understanding how films withstand surface challenges during handling or storage.

### 3.4. Functional Efficacy

#### 3.4.1. Antimicrobial Activity

Antimicrobial activity analysis can evaluate the ability of coatings to inhibit the growth of spoilage and pathogenic microorganisms, a critical feature for extending the shelf-life of perishable animal-derived products. This analysis typically involves in vitro and in vivo methods to quantify the inhibitory effects against common foodborne pathogens (e.g., Psychotropic aerobic bacteria and Mesophilic bacteria, *Pseudomonas* spp. [26], *E. coli*, *Salmonella* spp., *L. monocytogenes*, and *Staphylococcus aureus*, spoilage organisms (e.g., molds, yeasts) [84], and lactic acid bacteria (LAB) [85]. Key techniques include agar well diffusion method to determine zones of inhibition (ZOI) and obtain the antibacterial activity, as well minimum inhibitory concentration (MIC), minimum bactericidal concentration (MBC), and time-kill kinetic studies [86,87]. These methods assess the efficacy of the natural biopolymer alone and/or combined with bioactive compounds (e.g., plant extracts, essential oils, or nanoparticles) incorporated into coatings, which disrupt microbial cell membranes, interfere with metabolic pathways, or inhibit enzyme activity [84,87].

Considering the biopolymer effect, previous research has shown that they can reduce microbial proliferation in animal-derived food products. Alginate, typically used in the form of sodium alginate, is a naturally occurring polysaccharide obtained from brown algae (*Phaeophyceae*). It has gained attention as a promising material for developing alginate-based edible coatings, demonstrating effectiveness in preserving a wide range of perishable items such as fruits, vegetables, meat, poultry, and cheese. The inclusion of crosslinking agents like calcium chloride (CaCl_2_) has been reported to enhance the functional performance of these coatings, particularly by improving microbial inhibition and reducing physicochemical deterioration [9]. On the other hand, in relation to the polymer/natural additive incorporation effect, Al-Hilifi et al. [88] assessed that the antimicrobial efficacy of chitosan was improved by incorporating ginger (*Zingiber officinale*) essential oil into an active edible coating. The resulting formulation exhibited inhibitory effects against several bacterial strains, including *S. aureus*, *Bacillus subtilis*, *Streptococcus* spp., *E. coli*, *Salmonella* spp., and *Pseudomonas aeruginosa*.

In addition, recent innovations focus on controlled release mechanisms (e.g., ginger nanocomposites in hydrogel matrices) with antimicrobial properties over time also have been studied. However, challenges persist, especially considering interaction with food matrices such as lipids or proteins in animal-derived products that can reduce bioactive compounds availability as well regulatory and sensory constraints. High concentrations of antimicrobial agents can alter food flavor or face regulatory barriers. Therefore, future work should prioritize harmonizing testing standards, optimizing bioactive compound delivery, and evaluating long-term stability under commercial storage conditions [33]. For instance, Kanelaki et al. [26] found that edible gels enriched with rosemary (*Rosmarinus officinalis* L.) extract showed potential in prolonging the shelf-life of fish products by mitigating lipid oxidation. The addition of rosemary extract further suppressed lipid oxidation and TVB-N levels. Furthermore, the gels functioned effectively as carriers for the controlled delivery of the extract. However, authors reported a modest effect of this coating on limiting microbial growth for psychotropic and mesophilic bacteria, and *Pseudomonas* spp.

#### 3.4.2. Antioxidant Capacity

Antioxidant capacity analysis assesses the ability of coatings to scavenge free radicals and inhibit oxidative degradation, a critical factor in preserving the quality and safety of perishable food products. Oxidation of lipids and proteins in foods like meat, dairy, and seafood leads to rancidity, color loss, nutrient degradation, and shortened shelf-life [89,90]. Antioxidant activity is evaluated using in vitro assays such as total phenolic [mg gallic acid equivalent (GAE)/g], total flavonoids [mg quercetin equivalent (QE)/g], total flavonol (mg QE/g) contents as well DPPH and ABTS radical scavenging to measures the coating’s ability to neutralize stable free radicals (e.g., 2,2-diphenyl-1-picrylhydrazyl or 2,2′-azino-bis (3-ethylbenzothiazoline-6-sulfonic acid), respectively [91,92]. Similarly, FRAP (Ferric Reducing Antioxidant Power) quantifies the reduction of Fe^3+^ to Fe^2+^, indicating electron-donating capacity [21]. In addition, concerning the lipid oxidation tests, the thiobarbituric acid reactive substances (TBARS) or peroxide value (PV) assays track secondary oxidation products in coated foods during storage [23]. These methods validate the efficacy of antioxidants (e.g., polyphenols, vitamins, or plant extracts) integrated into coatings, which delay oxidative processes by donating hydrogen atoms, chelating pro-oxidant metals, or interrupting radical chain reactions.

Antioxidant analyses are essential for designing coatings that combat oxidative spoilage in animal-derived foods. Coatings enriched with natural antioxidants (e.g., tocopherols in gelatin films, rosemary extract in chitosan matrices, or curcumin in alginate coatings) are assessed to determine effective concentrations. For example, whey protein coatings with encapsulated green tea (*Camellia sinensis*) polyphenols show dose-dependent DPPH scavenging, correlating with reduced lipid oxidation in packaged meats [64]. Chitosan films enriched with bay tree (*Laurus nobilis*) and black pepper (*Piper nigrum*) essential oils demonstrated synergistic effects according to the additive included, lowering TBARS values in stored beef patties only for the *L. nobilis* treatment [3]. Moreover, real-time oxidative stability must be determined by storage trials under controlled conditions (e.g., light, temperature, oxygen exposure) to monitor antioxidant retention and activity.

Moreover, it is important to consider that polymers alone (e.g., chitosan) have already shown antimicrobial and antioxidant activity. For instance, previous studies have attributed chitosan’s antioxidant properties to its protonated amine groups, which are capable of stabilizing free radicals [93]. Considering the chitosan effect in food matrices, this polysaccharide has been shown to slow the development of black spots in shrimp (*Pandalus borealis*), primarily due to its ability to chelate metal ions and form a barrier that limits oxygen exposure, thereby inhibiting the activity of the polyphenol oxidase enzyme [32].

Lastly, recent advances in fluorescence-based techniques have significantly enhanced the evaluation of antioxidant capacity in polymeric coatings. Among these, the use of fluorescent probes such as boron-dipyrromethene (BODIPY) derivatives, has proven highly effective in detecting lipid peroxidation. This lipophilic probe allows to quantify and offer spatial assessment of oxidative degradation within biopolymer matrices. Its application has been especially useful in food-grade systems, such as electrospun zein fibers encapsulating corn oil, where BODIPY probes enabled real-time monitoring of oxidative processes [94]. These non-invasive assays provide sensitive and localized measurements of antioxidant performance in situ, making them particularly suitable for natural coatings applied to animal-derived products.

As discussed above, interaction between components is complex and antioxidants may interact with coating polymers, altering film solubility or mechanical properties or demonstrating environmental sensitivity to light or heat during processing, degrading labile antioxidants. For this reason, antioxidant capacity analysis bridges laboratory research and practical food preservation, ensuring coatings mitigate oxidative spoilage while aligning with sustainability goals. Future research should prioritize in situ validation of antioxidant efficacy, scalable encapsulation techniques, and harmonized testing standards to solve this issue.

### 3.5. Food-Specific Characterization and Food Matrix Compatibility

Food-specific characterization evaluates how natural polymer-based coatings interact with the physicochemical and biological properties of perishable animal-derived products, such as meat, dairy, and seafood. Food matrix compatibility refers to the ability of coatings to adhere to, function within, and remain stable under the unique conditions of each food type (e.g., pH, moisture content, lipid oxidation potential, microbial load). This analysis ensures coatings maintain their preservative functions, such as moisture retention, oxygen barrier efficacy, antimicrobial/antioxidant activity, without compromising sensory attributes (e.g., texture, color, flavor) or food safety [12,95].

Nowadays, the challenges of meat products regarding this topic are fresh meat’s high surface moisture and lipid oxidation characteristics that require strong adhesion, hydrophobicity, and antioxidant capacity coatings. For instance, some solutions have been proposed with protein-polysaccharide coatings like gelatin-chitosan films cross-linked with tannic acid to reduce the preservation of pork while inhibiting microbial growth. However, according to the authors [96], the coating demonstrated dual inefficacy: it failed to extend the shelf-life of the chilled pork and accelerated spoilage by acting as a nutrient-rich substrate that promoted microbial proliferation. Similarly, Vargas-Ramella et al. [9] evaluated the effects of sodium alginate coat cross-linked with CaCl_2_ on fish fillets (*Mugil liza*) during refrigeration, and found data contrary to prior reports about sodium alginate’s antioxidative effects. Authors observed that the CaCl_2_-cross-linked coating increased TBARS by 33% in the fish fillets, indicating a pro-oxidant activity in this food matrix. This contrast highlights how food composition (e.g., lipid profiles and enzymatic systems) critically determines coating efficacy.

Yet, concerning seafood, the main challenge is their inherent rapid microbial spoilage, TBARS, and TVB-N production that necessitate coatings with antimicrobial properties. In fact, conventional techniques to maintain seafood products’ quality (e.g., chilling) can be ineffective [26,91]. Solutions proposed suggested chitosan-gelatin coating alone to preserve shrimp quality (*Litopenaeus vannamei*) [32], xanthan coating embedded with propolis to preserve mackerel tuna (*Euthynnus affinis*) fillets [29], and muicle–chitosan for Cazon Fish (*Mustelus lunulatus*) fillets [86]. In these cases, coated samples proved to extend the shelf-life and maintain the quality of the shrimp and the fillets during refrigerated storage in terms of pH, peroxide value, TVB-N, TBARS, microbiological and sensory properties.

As discussed above, there is a matrix complexity due to the heterogeneous food surfaces (e.g., meat, dairy products and seafood) that challenge obtaining coating products with uniformity and adhesion. In addition, scalability issues such as lab-scale successes (e.g., dip-coating) may fail in industrial settings due to processing speed or cost constraints (e.g., spray-coating equipment for seafood). Finally, high concentrations of bioactive compounds (e.g., essential oils) may impart off-flavors or face regulatory restrictions for direct food contact. Consequently, food-specific characterization is critical to advancing natural polymer-based coatings from experimental prototypes to commercially viable solutions. Researchers can optimize adhesion, stability, and functional efficacy by tailoring formulations to the unique demands of meat, dairy, and seafood matrices. Innovations in responsive coatings and nanocomposites offer promising pathways to overcome compatibility challenges, though scalability and regulatory compliance. Future research should prioritize industrial-scale trials, consumer sensory studies, and harmonizing food-contact safety standards.

Therefore, in light of the aforementioned limitations, subsequent sections critically evaluate the efficacy of diverse natural polymers and additives within animal-derived food matrices. To elucidate their preservative mechanisms, these investigations assess dependent interactions between biopolymer formulations and substrate-specific variables.

## 4. Animal-Derived Products: Practical Applications and Mechanisms of Effects

**Figure 1:**
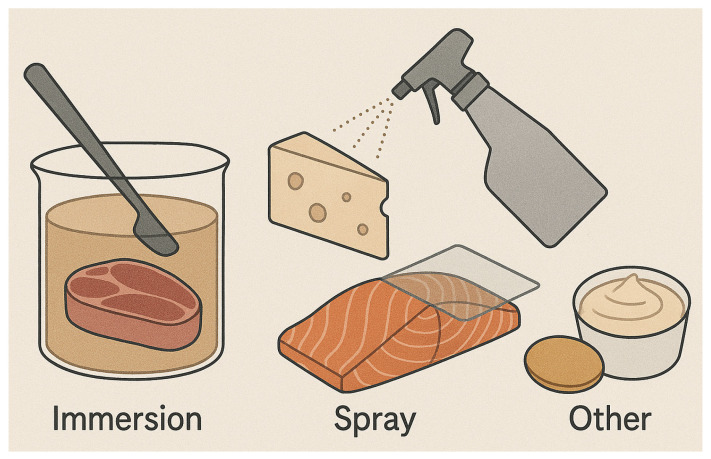
Coating applications in animal-derived food products.

Natural polymer-based coatings are applied to animal-derived products (meat, dairy, seafood, eggs) through techniques such as immersion (dipping), spray-coating, or brushing, using polysaccharide-, protein-, or lipid-based matrices (Figure 1). Advanced methods include encapsulation of bioactive compounds (e.g., essential oils) via nanocomposites or emulsions to enhance functional properties like antimicrobial activity and moisture retention. These coatings are tailored to specific food matrices through cross-linking or hybrid formulations, ensuring adhesion and stability under storage conditions. The efficacy of these coatings varies across product types due to differences in parameters such as surface composition, moisture content, and microbial load. The subsequent sections will critically evaluate their efficacy, challenges, and optimization strategies for each animal-based product category.

### 4.1. Fresh Meat and Meat Products

Fresh meat is a highly perishable product due to its high-water activity and rich macronutrient composition, which make it an ideal substrate for microbial proliferation. Moreover, its content of fat and proteins renders it particularly susceptible to lipid and protein oxidation, respectively [97]. Due to this perishable nature, fresh meat significantly contributes to food waste, as its shelf-life is markedly limited [98]. Furthermore, its production has a significant economic and environmental impact, making appropriate packaging essential to extend its shelf-life and minimize losses.

In this context, natural polymer-based coatings have emerged as promising tools, as their application has been shown to effectively prolong the shelf-life of various types of meat and meat products (Table 1). For instance, in fresh poultry meat, which is known for having one of the shortest meat shelf-life [99], various natural polymer-based coatings enriched with bioactive substances (such as polyphenols, plant by-product extracts, or probiotics including *Lactobacillus casei*, *Bacillus coagulans*, and *Lactiplantibacillus plantarum*) have proven effective in extending its durability [45,58,74,100]. This improvement is primarily attributed to the reduction of microbial growth and the delay in lipid oxidation, often due to the antimicrobial and antioxidant activity of the bioactive compounds, as well as the barrier effect provided by the edible coating itself. Furthermore, in this type of meat, film coatings have been shown not to compromise sensory attributes [70,101] and in some cases, they even enhanced the organoleptic properties by the end of the storage period [58].

Similarly, the use of natural polymer-based coatings in pork and their products (e.g., sausages) has also shown promising results during storage [107]. For example, chitosan–coix seed starch films incorporated with an optimal combination of ZnO nanoparticles (5%) and *Artemisia annua* essential oil (8%) improved meat preservation parameters by lowering TVC, and reducing TBARS and TVB-N levels [23]. Additionally, Qin et al. [96] reported that egg white-chitosan-pectin cross-linked with tannic acid-nisin coating reduced water loss in chilled minced pork and delayed changes in taste, texture, and surface color, while also lowering pH, TVB-N, carbonyl content, and microbial counts at the end of the storage period.

For fresh beef, the use of natural polymer-based coatings combined with bioactive agents such as essential oils significantly extended shelf-life by reducing microbial populations and lipid oxidation indicators, while maintaining acceptable sensory parameters. For example, the application of ginger essential oil extended the shelf-life of beef slices up to 9 days [102]. Similarly, coatings formulated with Shahri Balangu seed mucilage and 2% cumin essential oil extended meat shelf-life to 9 days by inhibiting bacterial growth and preventing increases in PV and MDA, while maintaining sensory attributes without negative effects [103].

Beyond fresh meat, natural polymer-based coatings have also been successfully applied to ready-to-eat (RTE) meat products, which are particularly susceptible to microbial contamination [108]. In this context, edible coatings act as physical barriers that reduce oxygen and moisture transfer and as protective matrices that inhibit microbial growth. For example, chitosan coatings applied to RTE beef meatballs significantly reduced the populations of *L. monocytogenes*, LAB, and *Enterobacteriaceae* during refrigerated storage, while maintaining acceptable sensory quality for up to 28 days (twice as long as uncoated controls) [105]. In a similar vein, Diao et al. [104] demonstrated that a nano-coating composed of carboxymethyl chitosan with garlic aqueous extract effectively delayed microbial growth and lipid and protein oxidation in spiced RTE chicken, without altering its appearance or taste.

### 4.2. Fresh and Processed Eggs

Eggs are highly consumed animal-derived products due to their high nutritional value, versatility, and affordability. However, they are also considered perishable goods, particularly fresh shell eggs, which are prone to quality degradation over time due to moisture loss, gas exchange through the shell, and microbial contamination [109]. Although they are naturally protected by a thin cuticle layer that acts as a barrier against microbial intrusion and moisture loss, this natural defense can be compromised during post-laying handling processes.

In some countries (such as Australia, Canada, Japan, and the United States), egg washing is routinely applied to reduce the surface microbial load and improve food safety. Nevertheless, this process could weaken the cuticle, increasing the risk of moisture loss and microbial penetration [110]. These alterations may lead to physicochemical changes such as pH increase, weakening of the albumen structure, and further deterioration of the cuticle, ultimately compromising the eggs’ freshness, safety, and sensory attributes [111]. To counteract these effects, natural polymer-based coatings have emerged as a promising alternative, with the potential to supplement or even replace the natural cuticle by forming an additional protective layer on the eggshell surface [112]. This strategy has been evaluated in various studies, revealing significant benefits in terms of egg preservation and shelf-life extension (Table 2). Pullulan-based coatings, either alone or combined with nisin, significantly reduced weight loss (below 1.5%) and helped preserve albumen and yolk quality for at least two additional weeks compared to uncoated eggs during storage at 25 °C, according to Morsy et al. [113]. Under refrigeration (4 °C), the improvement was even greater, as coated eggs maintained superior internal quality and a higher quality grade for four extra weeks. Moreover, although the addition of nisin did not lead to significant differences in weight loss, Haugh unit, or yolk index compared to pullulan alone, it proved advantageous in reducing microbial load during the 10-week storage period. In another study, Pan et al. [114] applied gellan gum emulsions containing rice bran oil and basil essential oil, which improved coating stability and barrier performance. Whether or not they included basil essential oil, eggs treated with these emulsions exhibited a slower decline in freshness indicators (Haugh unit, yolk index, and albumen pH) over 42 days of storage. Importantly, the inclusion of basil essential oil also contributed to reducing microbial growth.

Similarly to what has been observed in meat products, natural polymer-based coatings have been studied for fresh eggs and tested in processed eggs. In this regard, Venkatachalam et al. [27] investigated the effect of different coating materials (namely paraffin wax, chitosan, whey protein isolate, and soy protein isolate) on the quality changes of hard-boiled salted duck eggs stored at ambient temperature (30 ± 2 °C). Among the tested coatings, the whey protein isolate-based coating was the most effective, as it minimized weight loss, reduced microbial and lipid oxidation, and maintained better sensory attributes throughout 15 days of storage. For their part, Liu et al. [115] investigated an edible chitosan-based coating to preserve marinated eggs. They compared its effectiveness not only with uncoated marinated eggs, but also with chitosan coatings incorporating various bioactive compounds: ascorbic acid and tea polyphenols; nisin and ε-polylysine; and a combination of all four (ascorbic acid, tea polyphenols, nisin, and ε-polylysine). The results displayed that the combination of all four bioactives within the chitosan matrix was the most effective, as it significantly enhanced the stability of the marinated eggs in terms of pH, color, texture, and appearance. Furthermore, this formulation markedly inhibited microbial growth, extending the shelf-life from 12 to 20 days compared to uncoated samples.

### 4.3. Dairy Products

Dairy products are widely consumed worldwide in large quantities, primarily due to their high content of proteins, fatty acids, vitamins, and minerals [122]. Among them, cheese stands out as one of the most suitable candidates for applying natural polymer-based edible coatings, owing to its solid structure, which facilitates the formation of a protective coating, and its susceptibility to surface deterioration caused by moisture loss and microbial growth [85,123]. Furthermore, these coatings could replace conventional materials such as paraffin, offering a more sustainable alternative that could reduce weight loss and enhance the product’s microbiological, oxidative, and sensory stability throughout its shelf-life [124].

Given the variety of cheese types, natural polymer-based coatings have been tested on different cheese varieties (Table 2). For instance, El-Rahim et al. [116] investigated the use of whey protein concentrate-based antimicrobial edible films on soft cheese. Concretely, compared with an uncoated control, they examined the effect of incorporating sorbate, nisin, a combination of sorbate and nisin, or marjoram oil into the coatings. Their results showed that, although the differences between coated and uncoated cheeses were less pronounced in fresh samples, after 21 days of storage, the edible coatings led to improvements across all evaluated parameters (including reduced weight loss and microbial proliferation) with the most notable enhancement observed in sensory quality. The coatings containing the sorbate-nisin combination and those with marjoram oil were particularly effective. Similarly, Zaiden & Al-Hadidy [117] found that the preservation of soft cheese was improved by using a corn starch-based coating enriched with different concentrations (1–4%) of clove oil, compared to uncoated cheese. Although no significant differences were observed in moisture loss, the incorporation of clove oil effectively inhibited microbial growth and enhanced the sensory attributes throughout the 15-day storage period, particularly at clove oil concentrations of 3% and 4%.

The use of coatings based on sheep’s second cheese whey and whey protein isolate proved effective in preserving a model cheese made from cow’s milk [118]. These coatings, applied alone or supplemented with essential oils of oregano and clary sage, resulted in higher moisture content and water activity, as well as lower titratable acidity at the end of ripening (28 days), compared to uncoated cheeses and those coated with a commercial film containing natamycin. Although differences in color parameters were observed among treatments and during ripening, these differences were not significant at the end of the storage period. Regarding texture, the coatings reduced cheese hardness, chewiness, and gumminess. However, no improvements in microbial stability were detected compared to the commercial natamycin coating. In contrast, improved microbial safety was observed in semi-hard cheeses coated with chitosan films containing essential oils of oregano or rosemary [119]. In this study, Embuena et al. [119], also showed that the natural polymer-based coating effectively prevented weight loss and the degradation of lipids and proteins, with performance comparable to that of an antibiotic-based coating. From a sensory perspective, the oregano-containing coating applied twice stood out for preserving the cheese’s sensory attributes, whereas applying it three times (as well as the rosemary-based coating) led to a greater deterioration in sensory quality compared to the untreated cheese.

In the case of fresh cheese, edible coatings incorporating bacteriocin-producing LAB have shown promising results as a natural preservation strategy. Specifically, alginate-maltodextrin-glycerol coatings containing *Lactococcus lactis* L3A21M1 or *Lactococcus garvieae* SJM17, were effective in maintaining the viability and antimicrobial activity of the immobilized LAB cells throughout 10 days of storage at 4 °C and 10 °C. The application of these bioactive coatings reduced *L. monocytogenes* surface contamination and prevented its migration into the cheese matrix. Furthermore, the coatings inhibited the growth of spoilage mesophilic bacteria by day 6–8 of storage, while also reducing moisture and weight losses, without affecting pH or titratable acidity [120].

Additionally, natural polymer-based coatings have also been explored for the preservation of sliced cheese, a product particularly vulnerable to post-processing contamination due to handling. In this context, bilayer coating systems combining pullulan with either chitosan or gelatin, and enriched with hydrolats of lemongrass and curry plant, have shown promising results [121]. Both bilayer coatings (pullulan–chitosan and pullulan–gelatin) exhibited antimicrobial activity against *S. aureus*, attributed to the synergistic interaction between the incorporated hydrolats. However, the pullulan-chitosan coating demonstrated a more pronounced biocidal effect. In addition to their antimicrobial efficacy, these coatings enhanced the barrier and mechanical properties of the product, supporting their potential as multifunctional protective systems for sliced dairy products.

### 4.4. Seafood Products

Seafood products are highly perishable and susceptible to rapid deterioration due to their high moisture content, neutral pH, and abundance of free amino acids and unsaturated fatty acids. These characteristics make them especially prone to microbial growth, lipid oxidation, enzymatic activity, and other degradative reactions, significantly limiting their shelf-life and compromising their safety and sensory properties [125]. Consequently, developing effective preservation strategies, such as natural polymer-based coatings (Table 3), is essential to prolong the freshness and preserve seafood quality during storage and distribution [90]. Particular attention has been given to fresh fish meat, which is highly vulnerable to spoilage during chilled storage and therefore represents a key target for applying such preservation strategies. For instance, to reduce plastic use and extend the shelf-life of lebranche mullet fish fillets, Vargas-Ramella et al. [9] investigated the application of alginate coatings crosslinked with CaCl_2_. These coatings showed promising results by delaying moisture absorption, reducing pH, TVB-N formation, and microbial growth during refrigerated storage (12 days). However, a notable limitation was the increased lipid oxidation associated with the crosslinked alginate, highlighting the need to formulate natural polymer-based coatings enriched with antioxidant compounds.

For their part, Hager et al. [126] highlighted that, even with continued use of plastic packaging such as polyvinyl chloride (PVC) and vacuum packing, the application of edible coatings could significantly improve microbial control in fresh fish. Specifically, in hybrid striped bass fillets, zein-based coatings enriched with nisin or lemongrass essential oil demonstrated a reduction in *L. monocytogenes* growth during chilled storage. The strongest inhibitory effect was observed in nisin-coated samples, while PVC packaging provided the most effective conditions for both coatings. Significant effects related to the composition of the films were also reported by Xiong et al. [127]. After testing gelatin- and chitosan-based coatings, they observed that although gelatin alone effectively inhibited discoloration, pH variation, lipid and protein oxidation, and microbial growth in salmon fillets, it was less effective than chitosan-based films. Notably, the combination of both natural polymers provided superior performance. Furthermore, the incorporation of gallic acid enhanced the antioxidant properties of the coatings, while the addition of clove oil contributed to a stronger antimicrobial effect. When both bioactive compounds were included in the gelatin-chitosan matrix, the shelf-life of the coated salmon fillets was extended by at least five days compared to the uncoated control.

Despite the improvements achieved by incorporating bioactive ingredients such as essential oils, the organoleptic characteristics imparted to fish products must be carefully considered. Previous studies have reported that coatings incorporating these compounds can negatively affect the sensory attributes of frozen fish. For instance, Vieira et al. [129] found that although a chitosan-based coating containing clove oil effectively inhibited the growth of psychrotrophic bacteria and reduced both pH and moisture content in frozen tambaqui fillets after 120 days of storage, panelists preferred the sensory properties of uncoated samples or those coated with chitosan alone. Similarly, Berizi et al. [130] observed that, despite the promising antimicrobial activity of pomegranate peel extract, its use at higher concentrations (4%) resulted in undesirable colour changes in rainbow trout fillets. Therefore, a concentration of 2% was identified as the optimal level, ensuring microbial safety during storage while also improving textural properties.

## 5. Drawbacks, Research Gaps and Future Recommendations

Drawbacks: Despite their potential, natural polymer-based coatings face several limitations. A primary challenge is the inherent hydrophilicity of polysaccharide- and protein-based matrices, which compromises moisture barrier efficacy in high-humidity environments, limiting their applicability to moisture-sensitive products like fresh meat and seafood. Batch-to-batch variability in biopolymer sources—such as differences in chitosan deacetylation degrees or gelatin bloom strength—hinders reproducibility and scalability, creating inconsistencies in industrial applications. Furthermore, complex interactions between biopolymers, functional additives (e.g., essential oils) and food components (e.g., lipids, proteins) can let to undesirable effects such as accelerated lipid oxidation or microbial proliferation. Lastly, sensory drawbacks, including off-flavors from high concentrations of bioactive compounds, and regulatory constraints on direct food-contact materials further complicate commercialization.

Research Gaps: Critical knowledge gaps can affect the transition from lab-scale to real-world implementation. First, there is limited understanding of long-term coating stability under different storage conditions, such as temperature, light, or mechanical stress during storage and transportation. Second, environmental impact assessments are sparse; few studies evaluate the biodegradation kinetics of coatings in ecosystems (e.g., marine vs. terrestrial environments) or their carbon footprint. Third, consumer acceptance studies are scarce, particularly regarding texture or aroma changes in coated products. Additionally, the relation between coating formulations and heterogeneous food surfaces (e.g., meat cuts, cheese types) remains underexplored.

Future Recommendations: Future research should prioritize interdisciplinary strategies to address the abovementioned challenges. Material innovation is essential, focusing on hybrid systems (e.g., polysaccharide-lipid-protein composites or nanocomposites) to enhance hydrophobicity, mechanical resilience, and bioactive retention. Standardizing biopolymer extraction and purification protocols would also mitigate batch variability, while advanced encapsulation techniques (e.g., nanoemulsions) could optimize controlled release of antimicrobials or antioxidants. Integrating smart packaging technologies, such as pH-responsive films or oxygen-scavenging systems, may enable the dynamic preservation of specific food matrices. Industrially, collaborations are needed to optimize high-speed coating methods (e.g., spray-coating, dip-coating) and validate efficacy on a large scale. Finally, regulatory standards must harmonize safety limits for novel coatings, balancing innovation with consumer protection. By bridging these gaps, natural polymer coatings can achieve scalability, sustainability, and consumer acceptance, revolutionizing food preservation paradigms.

## 6. Conclusions

Natural polymer-based coatings represent a pivotal innovation in the sustainable preservation of animal-derived products. As the global food industry faces increasing demands for eco-friendly packaging and improved food safety, these biopolymeric systems offer a dual function: reducing reliance on petroleum-based plastics and extending the shelf-life of highly perishable products such as meat, seafood, dairy, and eggs. Polysaccharides (e.g., chitosan, alginate), proteins (e.g., whey, gelatin), and lipids (e.g., beeswax) provide structurally diverse and functionally matrices capable of forming films and coatings with desirable mechanical, barrier, and bioactive properties.

Including natural additives—such as essential oils, phenolic compounds, and bacteriocins—can enhance the coatings’ antimicrobial and antioxidant efficacy, inhibiting spoilage microorganisms and oxidative deterioration. In vitro and in vivo studies confirm that such coatings can significantly reduce microbial counts, limit lipid and protein oxidation, and preserve sensory attributes during storage.

However, despite their promise, the real-world application of these coatings remains limited by formulation challenges, matrix incompatibility, and industrial-scale limitations. The interaction between coating composition and food matrix is complex, and improper combinations may result in diminished performance or undesirable effects such as accelerated oxidation or textural impairment. Furthermore, regulatory approval, consumer acceptance, and cost-effectiveness must be addressed before widespread adoption can be realized.

Characterization techniques such as FTIR, SEM, TGA, tensile testing, and rheology have proven to be applied in understanding the structure-function relationships of the coatings. However, standardized testing protocols and real-world validation remain limited. Future advancements must prioritize developing matrix-specific formulations, harmonizing analytical methods, and integrating advanced technologies.

In conclusion, natural polymer-based coatings are promising strategies for sustainable food packaging innovation. Their continued development offers a route to enhance food security, minimizing environmental impact as a more natural food preservation solution. However, interdisciplinary collaboration between food scientists, material engineers, and industry will be essential to accelerate their transition from experimental concepts to viable commercial applications.

## Figures and Tables

**Table 1:** Application of natural polymer-based coating in meat and meat products.

Product	Edible Coating Main Composition	Main Effects of Natural Polymer-Based Coatings	References
Fresh meat			
Chicken breast	*Vicia villosa* protein isolate with ZnO NPs	Lower TVC, LAB, TBARS, and TVB-N levels; acceptable sensory attributes in coated fillets	[79]
Chicken breast	Xanthan gum-pectin with sweet orange peel EO	Minimized weight loss, lower TVB-N levels; coating biocompatible with L929 cells	[52]
Pork meat	Chitosan-coix seed starch with ZnO NPS *Artemisia annua* EO	Lower pH, TVC, TBARS, and TVB-N levels with the optimum combination	[23]
Minced pork	Egg white-chitosan-pectin cross linked with tannic acid-nisin	Reduced water loss and delayed changes in taste, texture, and color; lowered pH, TVB-N, carbonyl content, and microbial growth at the end of storage with the optimum combination	[96]
Beef slices	Sodium alginate-agar with ginger EO	Lowered microbial population; retarded lipid oxidation (reduced PV and MDA); extended shelf-life; and maintained acceptable sensory properties	[102]
Beef slices	Shahri Balangu seed mucilage with cumin EO	Extended meat shelf-life by inhibiting bacterial growth, reducing PV and MDA, with no negative impact on sensory attributes	[103]
Ready to eat meat			
Spiced chicken	Carboxymethyl chitosan with garlic acid extract	Reduced microbial growth and lipid and protein oxidation without affecting appearance or taste	[104]
Bovine meatballs	Chitosan	Reduced microbial growth and extended shelf-life up to 28 days without altering sensory characteristics	[105]
Carbonado chicken	Chitosan-gelatin with rosemary extract and ε-poly-L-lysine	Showed reduced TBC, mold and yeast counts, lipid oxidation, and pH changes, extending the shelf-life by at least 6 days under refrigeration	[106]

NPs: nanoparticles. TVC: total viable count. LAB: lactic acid bacteria. TBARS: thiobarbituric acid reactive substances. TVB-N: total volatile basic nitrogen. EO: essential oil. PV: peroxide value. MDA: malondialdehyde. TBC: total bacteria counts.

**Table 2:** Application of natural polymer-based coating in eggs and dairy products.

Product	Edible Coating Main Composition	Main Effects of Natural Polymer-Based Coatings	References
Eggs			
Whole fresh egg	Pullulan with nisin	Maintained internal quality, reduced weight loss, and extended shelf-life; nisin improved microbial growth control	[113]
Whole fresh egg	Gellan gum-rice bran oil emulsion with basil EO	Delayed freshness loss over 42 days and reduced microbial growth with basil EO	[114]
Hardboiled salted duck egg	Paraffin wax, chitosan, WPI, or SPI	WPI coating reduced weight loss, lipid oxidation, and microbial growth, and preserved sensory quality during 15 days at 30 °C	[27]
Marinated egg	Chitosan with ascorbic acid and tea polyphenols, or nisin and ε-polylysine (or their combination)	Chitosan coating with ascorbic acid, tea polyphenols, nisin, and ε-polylysine extended shelf-life of marinated eggs from 12 to 20 days, maintaining pH, color, texture, and reducing microbial growth	[115]
Dairy products			
Soft cheese	Whey protein with sorbate, nisin, both, or marjoram oil	Reduced weight loss and TBC, and improved organoleptic characteristics, with the coatings combining sorbate and nisin or those including marjoram oil standing out	[116]
Soft cheese	Corn starch with clove oil	Did not reduce moisture losses; microbial growth was inhibited and sensory characteristics were improved	[117]
Model cheese	SCW-WPI with oregano or clary sage	Increased moisture and water activity, reduced titratable acidity and texture parameters; no improvement in microbial stability over natamycin	[118]
Semi-hard cheese	Chitosan with rosemary and oregano essential oils	Increased microbial safety and reduced weight loss and lipid/protein degradation; in the case of oregano, aroma and flavor were improved (when applied twice)	[119]
Fresh cheese	Alginate with bacteriocin-producing *Lactococcus* spp.	Coatings with *Lactococcus* spp. reduced spoilage bacteria, *L. monocytogenes* and its migration; all coatings reduced weight loss without affecting pH or acidity	[120]
Sliced cheese	Pullulan-chitosan or pullulan-gelatin bilayer with lemongrass and curry hydrolats	Enhanced barrier properties and showed stronger antimicrobial effect against *S. aureus* (enhanced in the case of the combination with chitosan)	[121]

TBC: total bacteria count. SCW: sheep’s second cheese whey. WPI; whey protein isolate.

**Table 3:** Application of natural polymer-based coating in seafood.

Product	Edible Coating Main Composition	Main Effects of Natural Polymer-Based Coatings	References
Fresh fish			
Lebranche mullet fillets	Sodium alginate cross-linked with CaCl_2_	Reduced moisture absorption, pH decay, TVB-N, and microbial growth, but lipid oxidation increased	[9]
Hybrid striped bass fillets	Corn-zein with nisin or lemongrass EO	Nisin and lemongrass both inhibited *L. monocytogenes* growth, with nisin more effective; PVC outperformed vacuum packaging	[126]
Salmon fillets	Gelatin-chitosan with gallic acid and clove oil	The combination of the two natural polymer with gallic acid and clove oils showed the best performance on the fresh salmon fillet preservation and extended the shelf-life for at least 5 days	[127]
Rainbow trout fillets	Gelatin nanogel with nisin-thymol	The combination of nisin and thymol provided the most effective inhibition against *L. monocytogenes*	[128]
Frozen fish			
Hybrid striped bass fillets	Corn-zein with nisin or lemongrass EO	Nisin and lemongrass both inhibited *L. monocytogenes* growth, with nisin more effective; PVC outperformed vacuum packaging	[126]
Tambaqui fillets	Chitosan with clove essential oil	Delayed lipid oxidation, inhibited psychrotrophic bacteria; clove oil addition negatively affected sensory properties	[129]
Rainbow trout fillets	Chitosan with pomegranate peel extract	Decreased pH, TBARS, TVB-N, LAB and mold counts; improved texture, but negatively affected colour	[130]
Salmon fillets	Chitosan	Chitosan (1.5%) showed improved performance in maintaining color and controlling microbial contamination	[131]

EO: essential oil. TVB-N: total volatile basic nitrogen. PVC: polyvinyl chloride. TBARS: thiobarbituric acid reactive substances. LAB: lactic acid bacteria.
